# Refined Composite Multi-Scale Reverse Weighted Permutation Entropy and Its Applications in Ship-Radiated Noise

**DOI:** 10.3390/e23040476

**Published:** 2021-04-17

**Authors:** Yuxing Li, Bo Geng, Shangbin Jiao

**Affiliations:** 1 School of Automation and Information Engineering, Xi’an University of Technology, Xi’an 710048, China; liyuxing@xaut.edu.cn (Y.L.); 2200320014@stu.xaut.edu.cn (B.G.); 2 Shaanxi Key Laboratory of Complex System Control and Intelligent Information Processing, Xi’an University of Technology, Xi’an 710048, China

**Keywords:** multi-scale permutation entropy, ship-radiated noise, refined composite multi-scale reverse weighted permutation entropy, feature extraction

## Abstract

Ship-radiated noise is one of the important signal types under the complex ocean background, which can well reflect physical properties of ships. As one of the valid measures to characterize the complexity of ship-radiated noise, permutation entropy (PE) has the advantages of high efficiency and simple calculation. However, PE has the problems of missing amplitude information and single scale. To address the two drawbacks, refined composite multi-scale reverse weighted PE (RCMRWPE), as a novel measurement technology of describing the signal complexity, is put forward based on refined composite multi-scale processing (RCMP) and reverse weighted PE (RWPE). RCMP is an improved method of coarse-graining, which not only solves the problem of single scale, but also improves the stability of traditional coarse-graining; RWPE has been proposed more recently, and has better inter-class separability and robustness performance to noise than PE, weighted PE (WPE), and reverse PE (RPE). Additionally, a feature extraction scheme of ship-radiated noise is proposed based on RCMRWPE, furthermore, RCMRWPE is combined with discriminant analysis classifier (DAC) to form a new classification method. After that, a large number of comparative experiments of feature extraction schemes and classification methods with two artificial random signals and six ship-radiated noise are carried out, which show that the proposed feature extraction scheme has better performance in distinguishing ability and stability than the other three similar feature extraction schemes based on multi-scale PE (MPE), multi-scale WPE (MWPE), and multi-scale RPE (MRPE), and the proposed classification method also has the highest recognition rate.

## 1. Introduction

With the development and application of modern sensor technology, it is possible to record time sequences accurately for further research [1,2], especially in the fields of resource detection, ocean, environmental monitoring, security, medical diagnosis, etc. As important equipment in the ocean, the ship-radiated noise generated by the ship during navigation is a complex multi-source coupled noise source, which will cause unnecessary health effects to the crew inside the ship and the marine fauna outside the ship [3,4], and the research on ship-radiated noise is also of great significance for improving the comprehensive acoustic performance of the ship [5].

In recent years, more and more attention has been paid to the impact of ship-radiated noise on environment and ecology [6]. In 2012, Aglaia Badino and others proposed that it is necessary to evaluate and control the impact of noise emission from ships [7]. Bernardini Marco conducted experiments on the impact of noise from small ferries at different ports on citizens in 2019 [8]. In 2020, Luca Fredianelli characterized the sound power level and 1/3 octave band sound power spectrum of seagoing ships while moving at low speeds, and analyzed the transmission characteristics of the noise emitted by different types of seagoing ships at different ports [9]. In the same year, Marco Nastasi and others studied the effects of parameters such as minimum distance, speed, and draft on noise emission from a ferry and conducted a multiple regression analysis on them [10].

Entropy, as one of the parameters representing the state of matter in thermodynamics, is a powerful metric of the degree of chaos, complexity, or disorder in time series, such as permutation entropy (PE), approximate entropy (AE), sample entropy (SE), fuzzy entropy (FE), and their improved and multi-scale ones [11,12,13,14,15,16]. However, as a fast and powerful symbolization method, PE has attracted much attention from researchers due to the unique advantages of the advantages of high efficiency and simple calculation [17].

In 2001, Bandt and Pompe proposed PE in a pioneering paper [18]. Its applications can also be found in the fields of economics [19,20], mechanical engineering [21,22], and underwater acoustics [23,24]. On the aspect of economics, PE can reveal the inherent law of the Shanghai and Shenzhen Stock Exchanges [20]. On the aspect of fault diagnosis, a hybrid fault diagnosis model for motor bearing was put forward that integrated PE, ensemble empirical mode decomposition (EEMD), and optimized support vector machine (SVM), where PE is used to detect fault states of motor bearing [21]. On the aspect of underwater acoustic signal processing, PE and multi-scale PE (MPE) are first applied to complexity feature extraction of underwater acoustic signal in 2016 and 2017, respectively [23,24].

In 2013, Fadlalah Bilal and others proposed weighted permutation entropy (WPE) and applied it in the field of medical signal processing [25]. Compared with PE, WPE introduced amplitude information for the first time on the basis of PE and has the following advantages: (i) WPE can better capture the abrupt changes in time series by weighted calculation; (ii) at low signal-to-noise ratio (SNR), WPE also has better stability and robustness. Moreover, the application superiorities of WPE have been found in different fields. In [26], a bearing multi-fault diagnosis method was put forward integrated EEMD, WPE, and improved SVM ensemble classifier, where WPEs of the first several intrinsic mode functions (IMFs) are served as the fault feature vectors of bearing vibration signals. In [27], PE and WPE were used to analyze two types of resting state EEG from diabetics, and the results show that WPE can better discriminate between the amnestic mild cognitive impairment diabetics and normal cognitive function diabetics. In [28], WPE is first used in feature extraction of underwater acoustic signal combined with duffing chaotic oscillator (DCO) and complete EEMD with adaptive noise (CEEMDAN), and it can provide more accurate feature information than PE.

In 2017, Bandt proposed another PE for the second time, which we call reverse PE (RPE) [29]. RPE was originally used to identify different sleep stages based on EEG data in the medical field. Then, RPE has been rapidly applied in the field of underwater acoustic. In [30], noise reduction and feature extraction schemes for ship-radiated noise are proposed based on RPE and variational mode decomposition (VMD), RPE has stronger recognition ability to noise IMFs than PE, and RPE has better separability than PE as the complexity feature of IMF for ship-radiated noise. In [31], RPE combined with DCO was applied to detect the line spectrum frequency, the results show that RPE can be more accurate to extract line spectrum frequency of IMF for ship-radiated noise.

**Figure 1:**
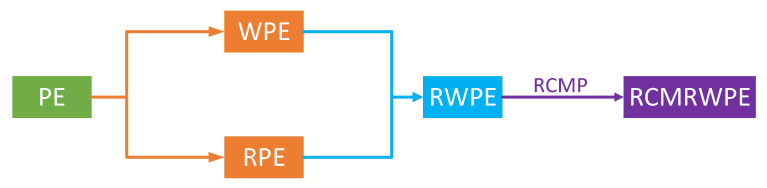
The origin of RCMRWPE.

In 2019, Li proposed reverse weighted PE (RWPE), which united the core ideas of WPE and RPE and has better inter-class separability and robustness performance to noise than PE, WPE, and RPE [32]. However, RWPE can only represent the complexity of signal under single scale, which cannot fully reflect the complexity of the signal and has some limitations. In order to break through these limitations, refined composite multi-scale RWPE (RCMRWPE) is put forward based on refined composite multi-scale processing (RCMP) and RWPE in this paper, and Figure 1 shows the origin of RCMRWPE. From Figure 1, it can be observed that WPE and RPE are the improvement of PE; RWPE combines the core technologies of WPE and RPE; RCMRWPE carries out refined composite multi-scale processing (RCMP) on the basis of RWPE, and RCMP is an improved method of coarse-graining, which not only solves the problem of single scale, but also improves the stability of traditional coarse-graining [33,34].

Most of the traditional feature extraction methods for ship-radiated noise are based on frequency or energy, for example, the aforementioned feature extraction schemes based on CEEMDAN and VMD, their superior performance in the extraction of ship-radiated noise has been confirmed in [35,36]. However, compared with these traditional feature extraction methods, the entropy-based feature extraction method can extract the complexity of the ship-radiated noise, and has better performance at distinguishing between different ships [14], such as PE, RPE, and WPE. In this paper, we apply RCMRWPE to the artificial random signals and actual underwater acoustic signals, and propose an underwater acoustic signal feature extraction scheme based on RCMRWPE. A large number of feature extraction and classification experiments for ship-radiated noise prove the superiority and effectiveness of the proposed feature extraction scheme.

In the next section, we introduce RCMRWPE in detail, and introduce the feature extraction scheme and classification method based on RCMRWPE, respectively. In Section 3, a large number of feature extraction and classification experiments are carried out to verify the effectiveness of the proposed feature extraction scheme in artificial random signals and ship-radiated noise classification. Section 4 summarizes the total research work.

## 2. Refined Composite Multi-Scale Reverse Weighted Permutation Entropy

RCMRWPE, as a novel complexity metric, is based on RWPE and RCMP. Therefore, this section first introduces RWPE, and then presents RCMRWPE using RCMP on the basis of RWPE.

### 2.1. RWPE

For a given time sequences $Y=\left\{y_{i},\;i=1,\;2,\;3,\;\ldots ,\;N\right\}$, it can be reconstructed as L vectors as follows:

(1) $$\left[\begin{array}{cccc} y_{1} & y_{1+\tau } & \cdots & y_{1+\left(m-1\right)\tau }\\ y_{2\;} & y_{2+\tau } & \cdots & y_{2+\left(m-1\right)\tau }\\ \cdots & \cdots & \cdots & \cdots \\ y_{j} & y_{j+\tau } & \cdots & y_{j+\left(m-1\right)\tau }\\ \cdots & \cdots & \cdots & \cdots \\ y_{L\;} & y_{L+\tau } & \cdots & y_{L+\left(m-1\right)\tau } \end{array}\right]$$

where τ and m represent the time delay and embedding dimension, and L=N−(m−1)τ.

The L vectors are rearranged according to the size as follows:

(2) $$y_{i+\left(j_{1}-1\right)\tau }\leq y_{i+\left(j_{2}-1\right)\tau }\leq \ldots \leq y_{i+\left(j_{m-1}-1\right)\tau }\leq y_{i+\left(j_{m}-1\right)\tau }$$

If $y_{i+\left(j_{a}-1\right)\tau }$ and $y_{i+\left(j_{b}-1\right)\tau }$ are equal for the same vector, which is defined as follows:

(3) $$y_{i+\left(j_{a}-1\right)\tau }<y_{i+\left(j_{b}-1\right)\tau },\;(a<b)$$

After that, all of the patterns for L vectors are as follows:

(4) $$\pi_{r}=\left(j_{1},\;j_{2},\;\ldots ,\;j_{m-1},\;j_{m}\right),\;\left(r=1,\;2,\;\ldots ,\;m!-1,\;m!\right)$$

**Figure 2:**
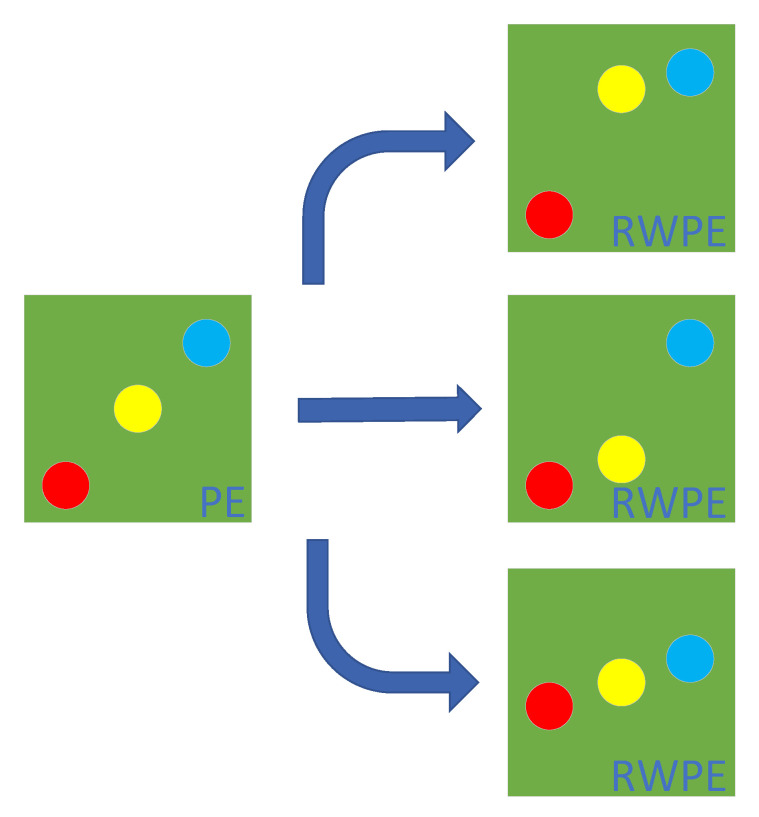
A pattern in PE and the corresponding three possible patterns in RWPW.

Due to the introduction of amplitude information, RWPE has more patterns than PE. For example, when m is 3, Figure 2 is a pattern in PE and the corresponding three possible patterns in RWPW.

For RWPE, the weight value of the pattern $\pi_{r}$ is represented as follows:

(5) $$\omega_{d}\left(\pi_{r}\right)=\frac{1}{m}\overset{m}{\underset{k=1}{\sum }}{\left(y_{\left(j+\left(k-1\right)\tau \right)}-{\overset{¯}{y}}_{j}\right)}^{2}$$

where ${\overset{¯}{y}}_{j}$ is the mean of the j-th vector, and it can be expressed as follows:

(6) $${\overset{¯}{y}}_{j}=\frac{1}{m}\overset{m}{\underset{k=1}{\sum }}y_{\left(j+\left(k-1\right)\tau \right)}$$

The frequency of the pattern $\pi_{r}$ can be expressed as:

(7) $$f\left(\pi_{r}\right)=\sum^{}f\left(\pi_{r,\;i}\right)\;\omega_{g}\left(\pi_{r,\;i}\right)$$

where $\pi_{r,\;i}$ is a possible pattern of $\pi_{r}$, $f\left(\pi_{r,\;i}\right)$ is the number of the pattern $\pi_{r,\;i}$. Thus, the probability of the $\pi_{r}$ can be represented as:

(8) $$P\left(\pi_{r}\right)=\frac{f\left(\pi_{r}\right)}{\sum_{r=1}^{m!}f\left(\pi_{r}\right)}$$

Like RPE, RWPE is represented as follows:

(9) $$H_{RWPE}=\overset{m!}{\underset{r=1}{\sum }}{\left(P\left(\pi_{r}\right)-\frac{1}{m!}\right)}^{2}=\overset{m!}{\underset{r=1}{\sum }}P{\left(\pi_{r}\right)}^{2}-\frac{1}{m!}$$

For example, there are two probabilities (p and 1−p), $H_{PE}\left(p\right)$, and $H_{RWPE}\left(p\right)$ are the function of p, which can be expressed as:

(10) $$\{\begin{array}{l} H_{PE}\left(p\right)=\left[-p\log p-\left(1-p\right)\log(1-p\right)]/\log2\\ H_{RWPE}\left(p\right)=2[{\left(p-\frac{1}{2}\right)}^{2}+\left(1-p-\frac{1}{2}{)}^{2}\right] \end{array}$$

**Figure 3:**
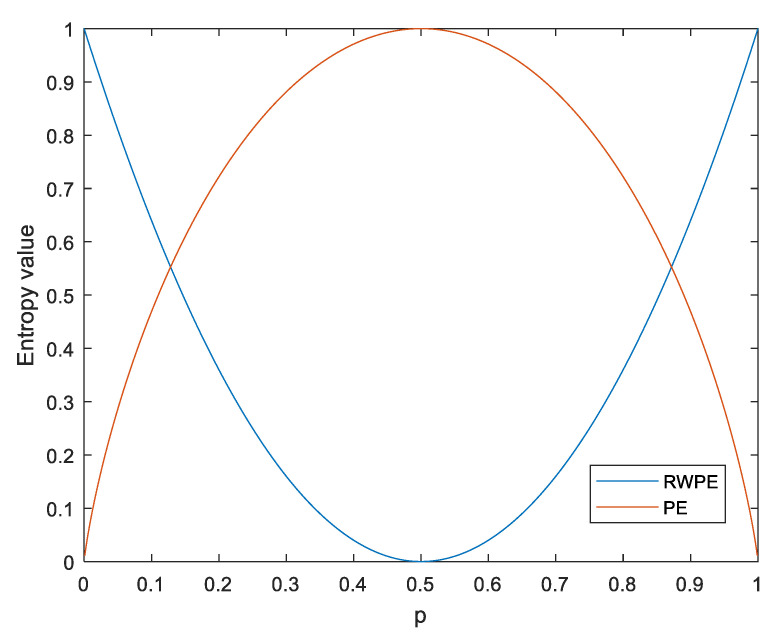
The functions $H_{PE}\left(p\right)$ and $H_{RWPE}\left(p\right)$.

Figure 3 shows the functions of $H_{PE}\left(p\right)$ and $H_{RWPE}\left(p\right)$. From Figure 3, it is evident that $H_{RWPE}\left(p\right)$ shows a reverse trend to $H_{PE}\left(p\right)$, $H_{PE}\left(p\right)$ and $H_{RWPE}\left(p\right)$ reach a maximum of 1 and a minimum of 0 when p is 0.5, and the two functions have the same range of values from 0 to 1.

**Figure 4:**
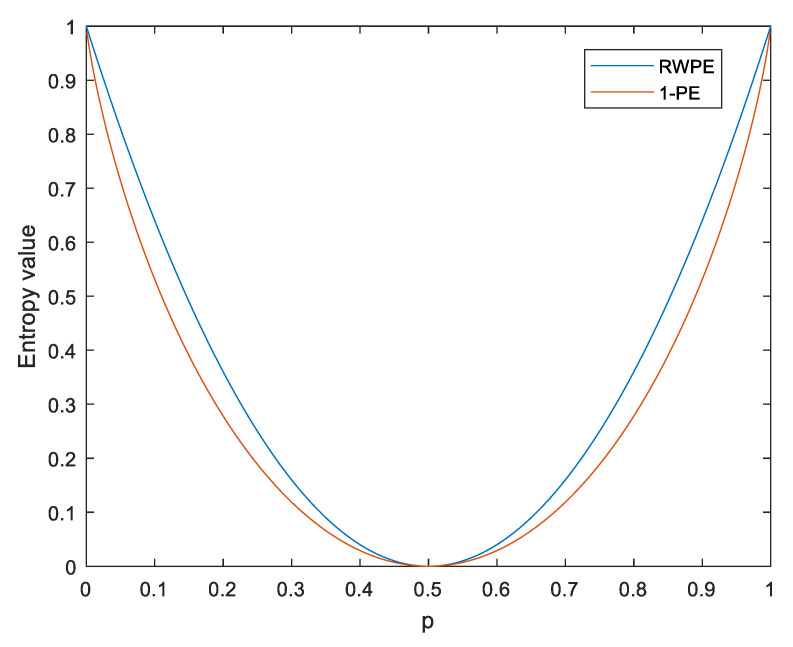
The functions $1-H_{PE}\left(p\right)$ and $H_{RWPE}\left(p\right).$

For ease of comparison studies, the functions $1-H_{PE}\left(p\right)$ and $H_{RWPE}\left(p\right)$ are shown in Figure 4. As seen in Figure 4, $1-H_{PE}\left(p\right)$ and $H_{RWPE}\left(p\right)$ have a similar varying tendency, and the two functions reach the maximum and the minimum at the same time. Therefore, like PE, RWPE can also be used to measure complexity.

### 2.2. RCMRWPE

For RCMRWPE, the RCMP of time sequences $X=\left\{x_{i},\;i=1,\;2,\;3,\;\ldots ,\;N\right\}$ can be shown as:

(11) $$Y^{\left(s\right)}=\left\{Y_{k}^{\left(s\right)},\;1\leq k\leq s\right\}$$

where s is the scale factor of RCMRWPE, $Y_{k}^{\left(s\right)}$ is the k-th result for RCMRWPE. Unlike coarse-graining for MPE, there are s results for RCMRWPE by RCMP, $Y_{k}^{\left(s\right)}$ can be represented as:

(12) $$Y_{k}^{\left(s\right)}=\left\{Y_{k,j}^{\left(s\right)},\;\left(1\leq j\leq \frac{N}{s}\right)\right\}$$

where $Y_{k,j}^{\left(s\right)}$ is can be represented as:

(13) $$Y_{k,j}^{\left(s\right)}=\frac{1}{s}\overset{k+js-1}{\underset{c=k+s\left(i-1\right)}{\sum }}x_{c}$$

**Figure 5:**
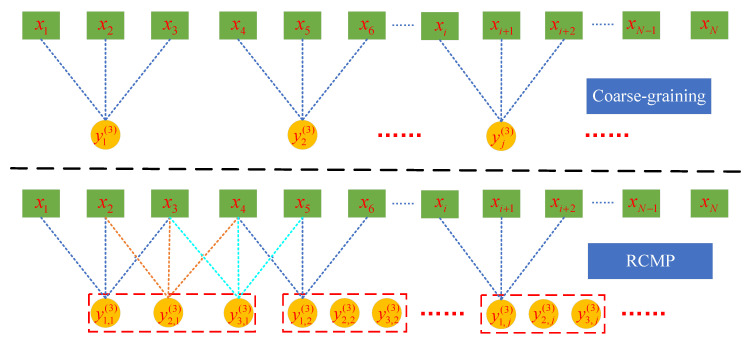
The coarse-graining for MPE and RCMP for RCMRWPE.

For instance, when embedding dimension m is 3, the coarse-graining for MPE and RCMP for RCMRWPE can be observed in Figure 5. As seen in Figure 5, RCMP can get more information from the original time sequences than the traditional coarse-graining.

Based on RWPE in Section 2.1, RCMRWPE can be defined as:

(14) $$H_{RCMRWPE}=\overset{m!}{\underset{r=1}{\sum }}{\left(\overset{¯}{P}\left(\pi_{r}\right)-\frac{1}{m!}\right)}^{2}=\overset{m!}{\underset{r=1}{\sum }}\overset{¯}{P}{\left(\pi_{r}\right)}^{2}-\frac{1}{m!}$$

where $\overset{¯}{P}\left(\pi_{r}\right)$ is the average value of $P\left(\pi_{r}\right)$ for $Y^{\left(s\right)}$.

### 2.3. The Feature Extraction Scheme and Classification Method Based on RCMRWPE

**Figure 6:**
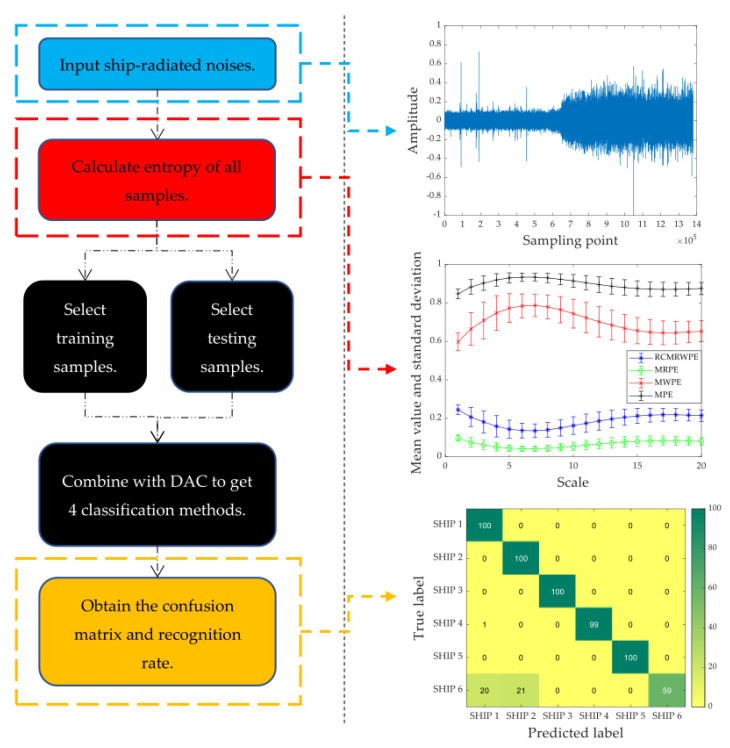
The flow chart of the proposed classification method.

In order to prove the good performance of RCMRWPE in ship-radiated noise classification, we take the RCMRWPE value under each scale as the extracted feature, and combine it with discriminant analysis classifier (DAC) to get a new classification method. In addition, we also combine MPE, MWPE, and MRPE with DAC, respectively, to get three classification methods, which are MPE, MWPE, and MRPE-based classification methods, and the effectiveness of the RCMRWPE based feature extraction scheme can be verified by comparing the classification recognition rate. Figure 6 shows the flow chart of the proposed classification method.

As shown in Figure 6, the details of the proposed classification method can be described as follows.

Step 1: First, we select several types of ship-radiated noise and draw their time-domain waveforms.

Step 2: Then, we calculate RCMRWPE, MPE, MWPE, and MRPE of all ship-radiated noise samples under 20 scales, and compare their mean and standard deviation (STD) entropy curves.

Step 3: Next, we combine the calculated entropy with DAC to form four classification methods, and randomly select training samples and testing samples to form training sets and testing sets.

Step 4: Finally, we calculate the recognition rate and confusion matrix of each classification method, which is used to verify the superiority of the proposed feature extraction scheme. In addition, we also carried out comparative experiments on different embedding dimension m to explore its impact on the classification performance of RCMRWPE.

## 3. A Ship-Radiated Noise Feature Extraction Scheme Based on RCMRWPE

### 3.1. Synthetic Signals

In this section, we introduce White Gaussian noise (WGN) and 1/f noise to verify the effectiveness of RCMRWPE as a feature extraction method. WGN and 1/f noise are two important signals to evaluate multi-scale entropy method. Generally speaking, the complexity of 1/f noise is higher than that of WGN, and the irregularity of WGN is higher than 1/f noise [17].

**Figure 7:**
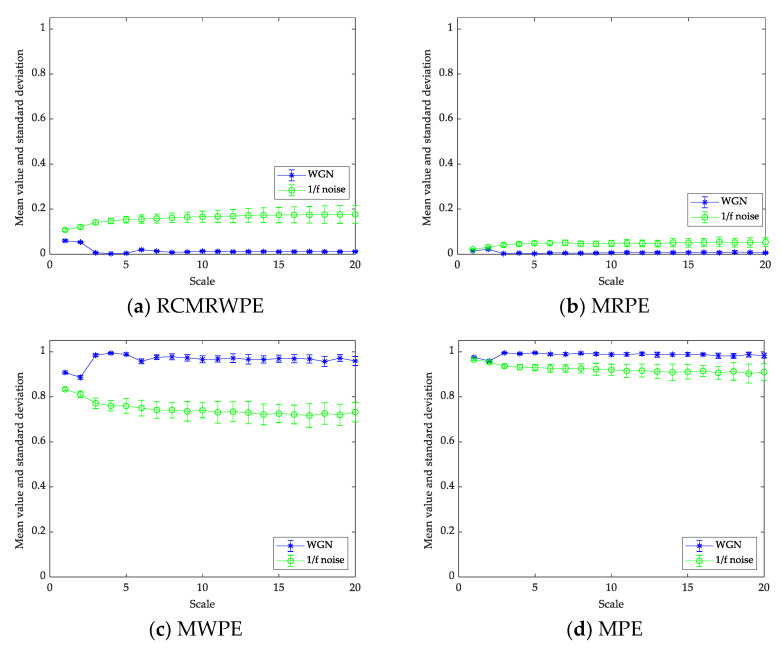
The mean and STD entropy curves of WGN and 1/f noise.

Figure 7 shows the mean and STD entropy curves obtained for RCMRWPE (m = 3), MRPE (m = 3), MWPE (m = 3), and MPE (m = 3) using 30 different 1/f noise and WGN signals with the length of 2000 samples. In addition, the time delay τ >1 will lead to the loss of frequency information, so we set τ as 1 in this paper. Comparing the four curves with different entropy in Figure 7, we can draw the following conclusions: (i) the RCMRWPE and MRPE value of WGN is larger than one of 1/f noise, which is opposite to the result of MPE and MWPE, this is because the reverse process is added to RCMRWPE and MRPE, which makes their results contrary to the fact, therefore, the four curves can all verify the conclusion that the irregularity of WGN is higher than 1/f noise, especially in RCMRWPE and MWPE curves; (ii) the STD of WGN under each curve is smaller than one of 1/f noise, and the difference between the STD of the two noises in the RCMRWPE and MWPE curves is relatively large, which is in line with the conclusion that the complexity of 1/f noise is higher than that of WGN; (iii) we can see that RCMRWPE and MWPE curves can distinguish signals more easily than the one of MPE and MRPE.

All these results indicate that the feature extraction scheme based on RCMRWPE and MWPE has a better performance than the other two schemes. In order to further compare their distinguishing ability and stability, we carry out many comparative experiments on ship-radiated noise.

### 3.2. Ship-Radiated Noise Datasets

**Figure 8:**
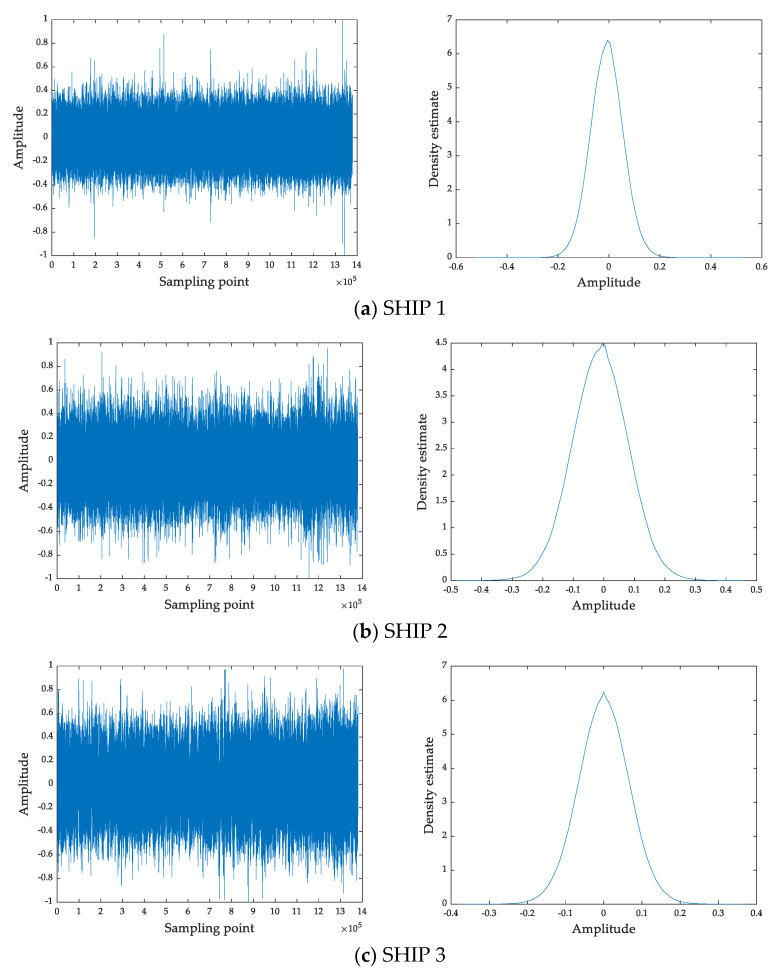

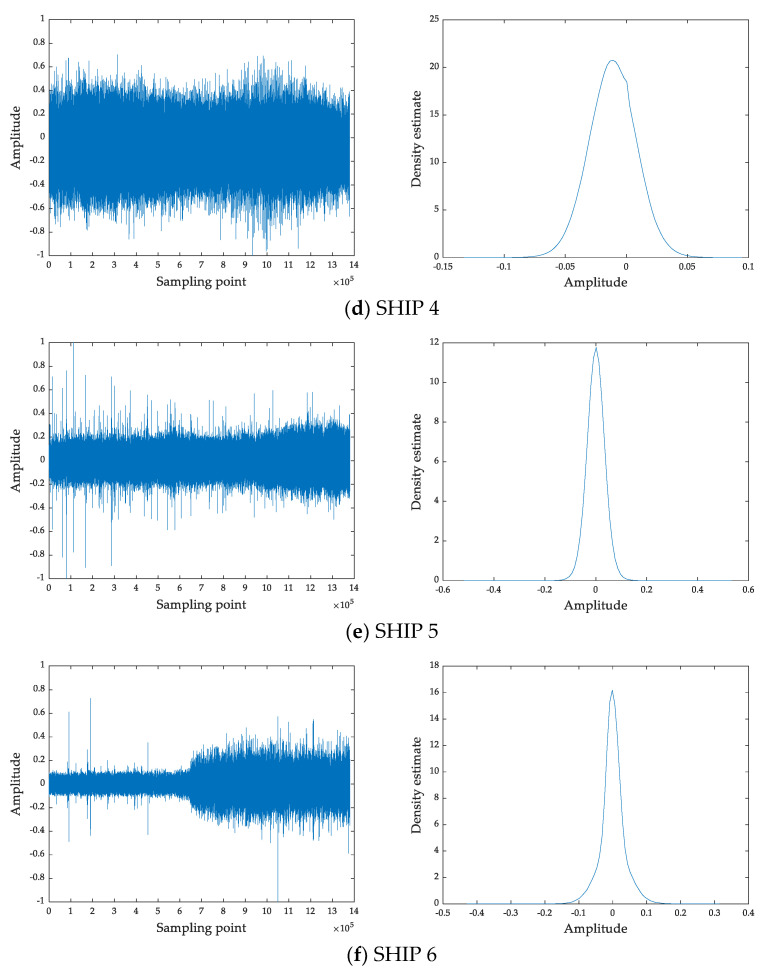
The normalized time-domain waveforms and probability density estimation function for six ship-radiated noise.

In this section, we select six ship-radiated noises to verify the validity of RCMRWPE. The six ship-radiated noises are termed SHIP 1, SHIP 2, SHIP 3, SHIP 4, SHIP 5, and SHIP 6, respectively. Table 1 shows the label information of ship-radiated noise experimental data. Each ship-radiated noise has 500 samples, and the length of each sample is 5000 points. The normalized time-domain waveforms and probability density estimation function for the six ship-radiated noises are shown in Figure 8, and the estimation of probability density is computed through using Matlab function ‘ksdensity.m’.

#### 3.2.1. Analysis of Feature Extraction

**Figure 9:**
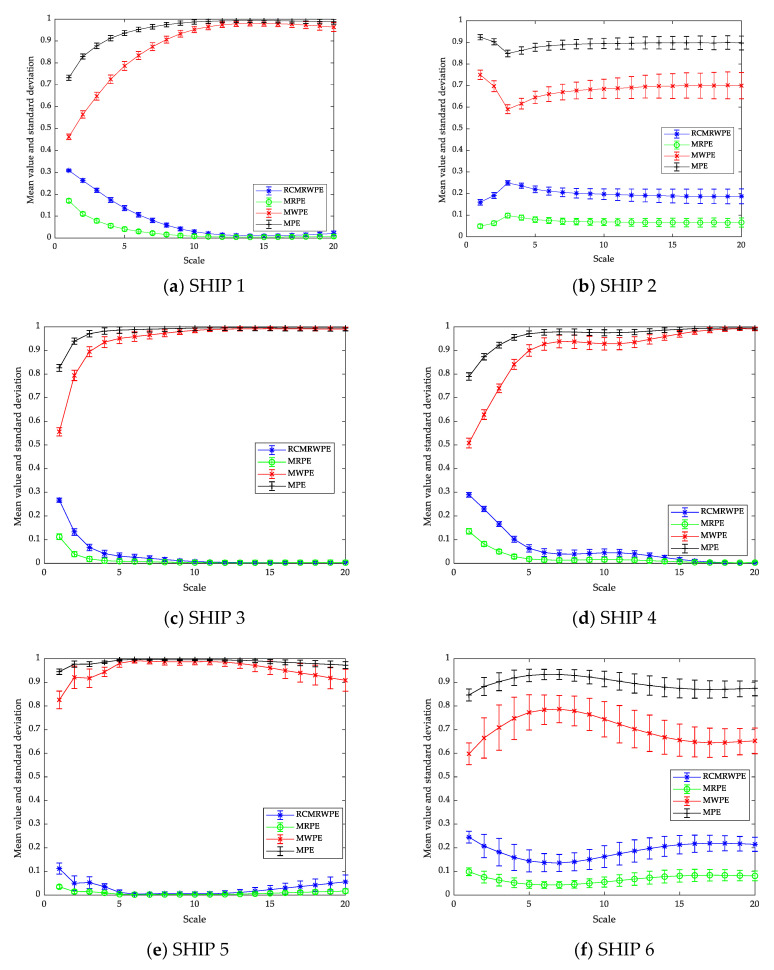
The mean and STD entropy curves of different ship-radiated noise.

First of all, we focus on testing the effect of feature extraction. Generally, the validity of RCMRWPE as a feature extraction scheme is judged by calculating the mean and STD RCMRWPE (m = 3) of six ship-radiated noise, and we apply MPE (m = 3), MRPE (m = 3), and MWPE (m = 3) with the feature number of 20 to the comparison of feature extraction. Figure 9 depicts the mean and STD entropy curves of six ship-radiated noise for different feature extraction schemes. From Figure 9, the STD of RCMRWPE and MRPE maintains a small value at each scale, which indicates that both of RCMRWPE and MRPE have a great stability in ship-radiated noise. We can also observe that MRPE and RCMRWPE curves share a similar trend, which is greatly different from that of MPE and MWPE curves.

#### 3.2.2. Analysis of Single Scale

From Figure 9, we can only get the mean and STD entropy. In this section, we use a discriminant analysis classifier (DAC) as a classifier and combine it with MPE (m = 3), MWPE (m = 3), MRPE (m = 3), and RCMRWPE (m = 3), respectively, to get four entropy-based classification methods. By comparing the recognition rate of each classification method under single scale, we can prove the effectiveness of RCMRWPE as a feature extraction scheme.

**Figure 10:**
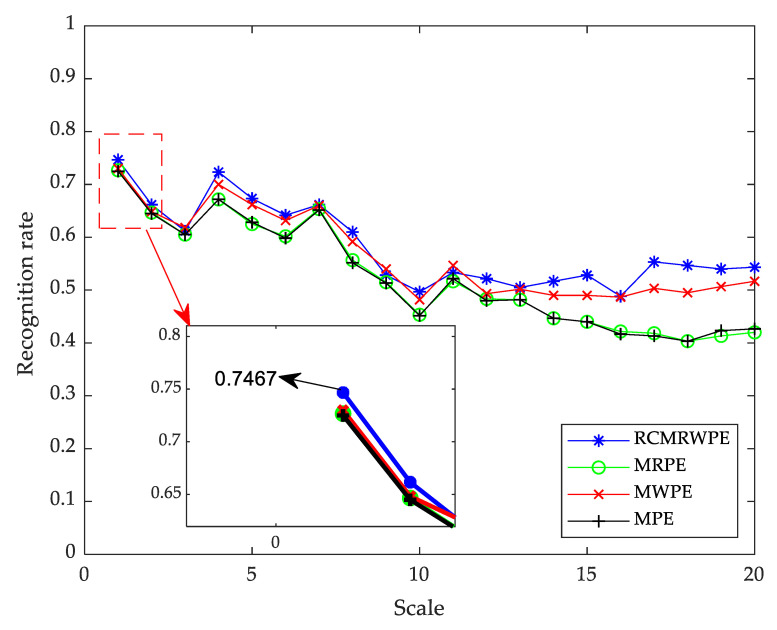
The recognition rate of four feature extraction schemes based on DAC.

Under each scale, 50 randomly selected samples of each ship-radiated noise are used as the training set, and 100 randomly selected ones are taken as testing set. Figure 10 shows the recognition rate of four feature extraction schemes based on DAC under 20 single scales. From Figure 10, it can be observed that when the scale is 1, each classification method has the highest recognition rate, among which RCMRWPE reached the highest value (0.7383). In addition, the classification method based on RCMRWPE has a higher recognition rate under most single scales than other classification methods, and with the increase of scale, the overall recognition rate presents a downward trend. The above results indicate that the proposed RCMRWPE-based classification method have much better performance than that of MRPE, MPE, and MWPE-based classification methods under a single scale.

#### 3.2.3. Analysis of Multi-Scale

In Section 3.2, the experimental results show the effectiveness of the classification method based on RCMRWPE, but the highest recognition rate is only 0.7383, which is due to the large number of noise categories and the few selected features. In order to further verify the good performance of the proposed feature extraction scheme in ship-radiated noise, in this section, we sequentially increase the number of selected features.

For comparison purposes, we, respectively, calculated the recognition rate of the MPE-based (m = 3), MWPE-based (m = 3), MRPE-based (m = 3), and RCMRWPE-based (m = 3) classification method under different numbers of features. Assuming that the number of selected features is n, like the experiment in Section 3.2, 50 randomly selected samples are used as the training set with dimension (50×n), and the testing set consisting of 100 samples with dimension (100×n). Table 2 shows the recognition rates of each classification method under various feature combination. In Table 2, the blue numbers are the lowest recognition rate of each classification method, and the red numbers are the highest recognition rate of each classification method. By observing their position distribution, it is evident that with the increase of the number of features, the recognition rate of each classification method is increasing, especially in RCMRWPE-based classification method, when the number of features is 13, its recognition rate is much higher than the other three classification methods, and has maintained a good performance in more feature combinations.

**Figure 11:**
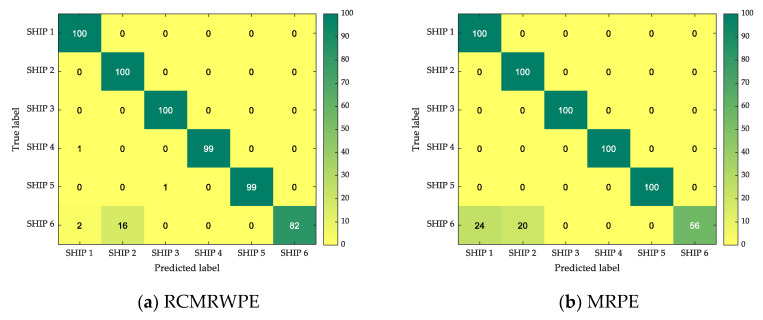

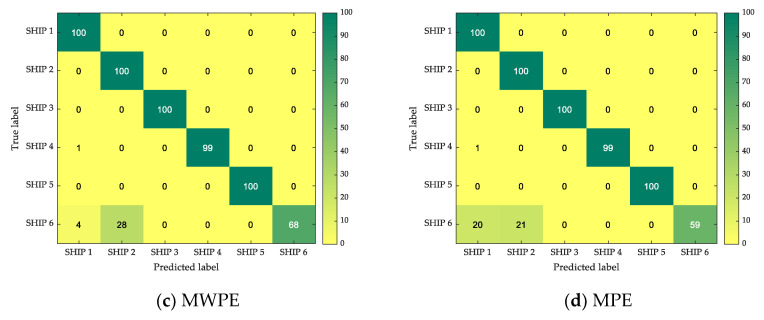
The confusion matrix under the combination of features with the highest recognition rate for each classification method.

From Table 2, we can also observe that the highest recognition rate of the RCMRWPE-based classification method is 0.9667, which has the best performance among four classification methods under 20 feature combinations. Figure 11 depicts the confusion matrix under the combination of features with the highest recognition rate for each classification method. By observing Figure 11, we can find that all the classification methods have a good classification performance for all selected ship-radiated noise except SHIP 6, and it is valuable to note that the main gap between different classification methods is reflected in the classification of SHIP 6. The RCMRWPE-based classification method has a recognition rate of 0.82 on SHIP 6, while the recognition rate of the other three classification methods on the SHIP 6 is only 0.56, 0.68, and 0.59, respectively, which states that the proposed feature extraction scheme has better performance in distinguishing ability than the other three feature extraction schemes.

#### 3.2.4. Analysis of Parameter Selection

In order to assess the sensitivity of RCMRWPE to the embedding dimension m, we selected different embedding dimension m for comparative experiments. For a clear comparison, we use the same training samples and test samples as in Section 3.2.3, and calculate the recognition rates of different classification methods with m = 4 and 5, respectively. It can also be seen from Table 2 that the highest recognition rate of 4 classification methods can be achieved with the number of features is greater than 15, therefore, in this section, we only compare the classification performance when the number of features exceeds 15. The details can be observed in Table 3. From Table 3, it is evident that compared with other classification methods, the classification method based on RCMRWPE has a higher recognition rate no matter when m = 4 or m = 5, which indicates that the change of m will not affect the fact that RCMRWPE has excellent performance in distinguishing ability and stability. In addition, we can also find that with the increase of m, the recognition rate of each classification method is also decreasing, this is because some information will be lost when m is too large, which may result in a poor classification performance.

## 4. Conclusions

In this paper, RCMRWPE is proposed by combining RCMP and RWPE, which aims to overcome the shortages of traditional multiscale distribution entropy in complexity measures of time series. Additionally, RCMRWPE is used in feature extraction of ship-radiated noise, through the comparison experiments of feature extraction and classification, the conclusions are included as follows:

(1) The feature extraction scheme based on RCMRWPE has smaller variance and better stability than the feature extraction scheme based on MWPE, and MPE.
(2) In the comparative experiment of different classification methods, the RCMRWPE-based classification method shows much better performance than MPE, MWPE, and MRPE-based classification methods under most single scales.
(3) In the multi-scale feature comparison experiment, when the number of selected features exceeds 15, each classification method has a good classification performance. Among them, the classification method based on RCMRWPE has the highest recognition rate, reaching 0.9667, which is 4% higher than the classification method based on MRPE.
(4) The increase of embedding dimension m will cause the loss of information in ship-radiated noise, but the RCMRWPE-based classification method still has a good classification performance, which further proves the distinguishing ability and stability of the proposed feature extraction scheme.

## Figures and Tables

**Table 1:** Label description of the used data.

Ship-Radiated Noise Class	Used Data
SHIP 1	State Ferry
SHIP 2	Cruise Ship
SHIP 3	Freighter
SHIP 4	Small Diesel Engine
SHIP 5	Motorboat
SHIP 6	Ocean Liner

**Table 2:** The recognition rate of each classification method for different numbers of features.

Number of Features	Recognition Rate			
	RCMRWPE	MRPE	MWPE	MPE
1	0.7467	0.7267	0.7300	0.7250
2	0.7733	0.8333	0.7767	0.8317
3	0.8300	0.8783	0.8317	0.8783
4	0.8333	0.8833	0.8283	0.8883
5	0.8300	0.8850	0.8317	0.8850
6	0.8300	0.8817	0.8317	0.8817
7	0.8317	0.8783	0.8267	0.8783
8	0.8333	0.8800	0.8267	0.8800
9	0.8333	0.8767	0.8317	0.8750
10	0.8333	0.8783	0.8283	0.8750
11	0.8400	0.8683	0.8283	0.8683
12	0.8667	0.8833	0.8300	0.8833
13	0.9533	0.8950	0.8567	0.8950
14	0.9650	0.9100	0.8933	0.9100
15	0.9617	0.9167	0.9383	0.9150
16	0.9650	0.9217	0.9433	0.9250
17	0.9667	0.9250	0.9450	0.9300
18	0.9600	0.9250	0.9367	0.9300
19	0.9617	0.9250	0.9367	0.9300
20	0.9600	0.9267	0.9367	0.9317

**Table 3:** The recognition rate of each classification method with different embedding dimensions.

Number of Features		16	17	18	19	20
Recognition Rate (m = 4)	RCMRWPE	0.9650	0.9650	0.9617	0.9633	0.9633
	MRPE	0.9183	0.9200	0.9200	0.9217	0.9167
	MWPE	0.9350	0.9367	0.9367	0.9383	0.9383
	MPE	0.8483	0.8483	0.8450	0.8500	0.8550
Recognition Rate (m = 5)	RCMRWPE	0.9217	0.9233	0.9200	0.9233	0.9250
	MRPE	0.9017	0.9017	0.9017	0.9017	0.8967
	MWPE	0.9050	0.9067	0.9083	0.9100	0.9083
	MPE	0.8433	0.8417	0.8433	0.8400	0.8400

## Data Availability

The data used to support the findings of this study are available from the corresponding author upon request.
